# Vertical Discrepancy in Height of Morse Cone Abutments Submitted to Different Torque Forces

**DOI:** 10.3390/ma14174950

**Published:** 2021-08-30

**Authors:** Bruno Q. S. Cordeiro, Carlos Fernando de Almeida Barros Mourão, Waldimir R. Carvalho, Edgard M. Fonseca, Pietro Montemezzi, Kayvon Javid, Cintia C.P. Martins, Valquiria Quinelato, Mylena D. Moreno, Priscila L. Casado

**Affiliations:** 1 Graduate Program in Dentistry, Universidade Federal Fluminense, Niteroi 24020-140, RJ, Brazil; brunoqsc@gmail.com (B.Q.S.C.); onecure@aol.com (K.J.); 2 Clinical Research Unit of the Antonio Pedro Hospital, Universidade Federal Fluminense, Niterói 24033-900, RJ, Brazil; 3 Clinical Research Laboratory in Dentistry, Universidade Federal Fluminense, Niteroi 24020-140, RJ, Brazil; 4 Implant Dentistry Department, Dentistry School, Universidade Federal Fluminense, Niteroi 24020-140, RJ, Brazil; wcarvalho@id.uff.br (W.R.C.); edgardfonseca@vm.uff.br (E.M.F.); ccpm.odonto@gmail.com (C.C.P.M.); valquiriaquinelato@yahoo.com.br (V.Q.); mylenamoreno@id.uff.br (M.D.M.); 5 Independent Researcher, 24128 Bergamo, Italy; m.montemezzi@libero.it

**Keywords:** morse cone, torque, abutment, implant-supported prosthesis

## Abstract

The present study aimed to evaluate the influence of manual torque (10 Ncm) versus clinical torque (30 Ncm), which is recommended by the manufacturer, on the total length of morse cone implant abutments. Twenty specimens were prepared and distributed into two groups: group 1 with ten analogs for morse cone type implant, and group 2 with ten morse type implants, size 4.3 × 15 cm. In each group, the distance between the implant platform to the top of the prosthetic abutment (abutment height) was measured and subjected to a torque of 10 Ncm. Then, the 30 Ncm torque was applied to the same abutment, and abutment height was measured. The distance between the top of the abutment and the implant/analog base was measured. In order to verify the clinical reproducibility of the experiment, comparisons between the abutment height of the analog at 10 Ncm and the implant at 30 Ncm were performed, showing a greater discrepancy in torque for the 10 Ncm analog (*p* < 0.05). In order to verify if the change in the laboratory protocol from 10 to 30 Ncm could minimize the differences in the height of the prosthetic abutments, the abutment height in groups 1 and 2 was compared with 30 Ncm, and no significant difference was observed (*p* > 0.05). The data indicated that the manual torque and the torque recommended by the manufacturer influence the total length of the prosthetic abutments of morse cone implants.

## 1. Introduction

Among many factors, the success of implant-supported rehabilitations depends on the configuration of the connection between the abutment and the implant platform, as it influences the stability of the prosthesis, the mechanical strength of the implant, and the prosthetic components [1]. For this, the professional should be aware that clinical procedures such as molding, transfer seating, and overload applied to the connection can lead to changes in the implant components which, as consequence, trigger modification in peri-implant tissues, decreasing implant success in the long term [2].

In the lab and oral cavity, the abutment is connected to the implant by a manual torque, while at the final prosthesis connection, the abutment will be connected to the implant to the proper torque value, which is the value of torque recommended by the manufacture [3]. Improper connection may cause interference that results in a lack of stability and prosthetic functionality. Therefore, the fit of an implant abutment superstructure, without any interference from manual torque during prosthesis confection to final torque, is essential to the success of the prosthesis [3].

In this context, a source of error when using an internal implant connection could arise from the level of the tightening torque applied to the prosthesis parts by the laboratory technician compared to that applied by the clinician in oral cavity [3]. However, this topic is not clearly addressed in the current literature, and information on the laboratory and clinical differences of prosthetic components of implants is still limited, requiring further studies in order to clarify and minimize prosthesis misfit.

All connections are known to have an amount of maladaptation and bacterial infiltrate, varying according to the type of implant platform [4]. However, the morse cone system seems to behave better, presenting excellent adaptation and less bacterial invasion when compared to other implant systems [4]. This is because the morse cone system manufacturers recommend the installation of the underlying implant at the level of the bone crest, which, usually, allows the maintenance of the peri-implant bone above the implant-abutment junction, even after loading [5]. The implant–abutment interface for morse cone connection could have a minimal or an absence of gap. This allows for bone growth in this space in the implant–abutment interface, establishing the contact between bone and implant during the stabilization of the implant pillar [6], which can reduce future damage to peri-implant tissue.

Since crown misalignment and adaptation between implant and abutment could impact the clinical performance of implant-supported prosthesis, it is up to the professional to understand the stages of the screw–implant prosthesis connection [7]. To minimize the screw loosening, it is documented in the literature that the screw should be retightened at least twice at 10-min intervals in all laboratory and clinical procedures [8]. For this, the use of specific wrenches is indicated along with the specific mechanical torque, following the manufacturer’s indications for the type of prosthetic abutment [9]. However, this procedure in the laboratory phase is usually not feasible. The need to remove the prosthetic component for clinical installation may cause physical changes and discrepancy of forces applied during implant torque and the tightening process [10].

Adaptation of the implant-supported prosthesis is crucial for the longevity of endosseous implant rehabilitation treatment. There are many factors that may interfere with prosthetic stability that can directly influence the development of future clinical protocols. Therefore, it is important to determine the vertical discrepancies between non-torqued and torqued abutments, since it is directly associated with occlusal contact adjustments in implant crowns and implant success in the long term. However, the scarcity of studies regarding clinical and laboratory aspects focusing on forces applied to prosthetic connection can lead to a gap in the basic knowledge related to making and adaptation of the prosthesis. Therefore, the objective of this study is to evaluate the discrepancy in axial displacement of morse cone implant abutments when compared to manual torque in the laboratory with that recommended by the manufacturer in oral cavity. Our hypothesis is that after torque application during prosthesis confection, there are discrepancies in the abutment implant complex.

## 2. Materials and Methods

### 2.1. Research Design

This research is of a technical laboratory nature, not involving the participation of humans or animals, or any procedure that includes biological material or personal data; therefore, it is not necessary to submit this research for evaluation by the Research Ethics Committee.

Research Instrument: In this study, 20 specimens were made for further division into 2 groups: group 1 with 10 analogs for morse cone implant type (Conexão, Implant Systems, São Paulo, Brazil), numbered 1 to 10; and group 2 with 10 implants, morse cone type, with size 4.3 × 15 cm (Conexão, Implant Systems, São Paulo, Brazil), numbered from 11 to 20.

All implants or analogs were installed using the same methodology: inside resin cylinders, density 1.42 g/cm^3^, with elastic modulus greater than 3 GPa (Polyacetal, Caterplast, São Paulo, Brazil) in type IV durone stone plaster (Dentsply, Dentsply Ind. Com., Rio de Janeiro, Brazil).

**Figure 1:**
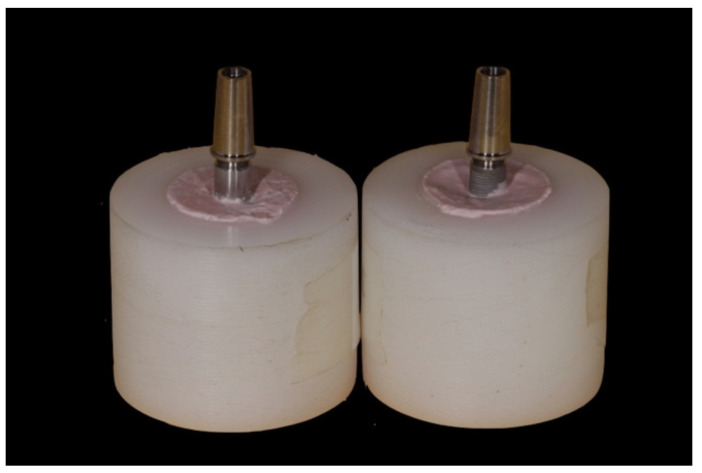
Resin cylinder to standardize the fixation of analogs (**left**)/implants (**right**).

A 7 mm diameter and 17 mm deep guide hole was drilled into the resin cylinder to standardize the fixation of implants/analogs and the amount of plaster used so that implants and analogs would not move when the abutment attached to them was torqued (Figure 1). Twenty 9.0 mm preparation abutments (Conexão, Implant Systems, São Paulo, Brazil) screwed with the Torq Control^®^ universal torque wrench (Anthogyr PT, Sallanches, France) were used on each implant and analog of both groups.

**Figure 2:**
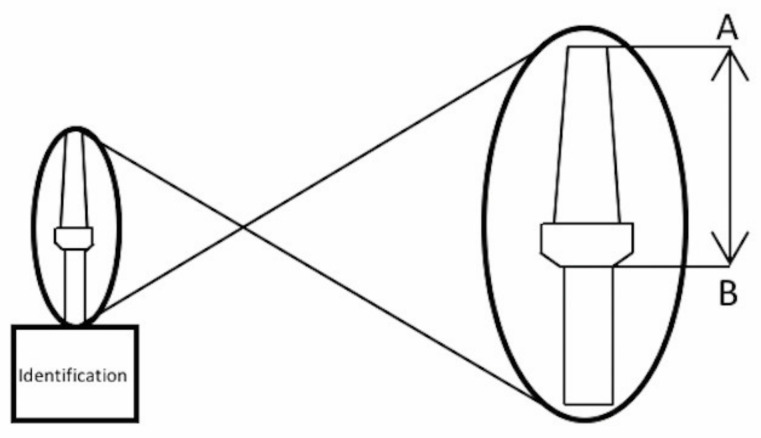
A–B distance (mm). A: A–B distance (distance between the implant platform to the top of the abutment). The letter A represents the maximum height of the abutment and the letter B represents the base of the implant.

Each pillar was subjected to the same torque force between the groups: 10 Ncm and 30 Ncm, presenting at the end the following division of groups: group 1 (analog; n = 10): 10 N and 30 N; group 2 (implant; n = 10): 10 Ncm and 30 Ncm. In each group, the abutment was subjected to the torque of 10 Ncm, and the first distance A–B was measured. Then, the 30 N torque was applied to the same abutment, and the second measurement A-B was made (Figure 2).

The distance between the top of the abutment and the implant/analog base (A–B) was measured at the Brazil Mitutoyo Calibration Laboratory, accredited by CGCRE according to ABNT NBRI ISO/IEC 17025, under number CAL0031, with a measurement uncertainty of 0.003 mm, a coverage factor (k) of 2.00, and infinite degrees of freedom.

### 2.2. Statistical Analysis

The measurements obtained in groups 1 and 2, with different torques of 10 N and 30 N, were tabulated and subjected to qualitative statistics, considering the median and standard deviation of the numerical variables evaluated. Microsoft Office 2013 Excel was used for data tabulation and Prisma GraphPad 6.0 software was used for statistical calculations and graphic production. The sample size was based on previous studies [7,8], considering the power of 80%.

The paired *t*-test correlated the measurements of the variables studied within each group, after verifying the distribution normality, with a significance level of 0.05%. Values of *p* < 0.05 were considered significant.

## 3. Results

The results considered the statistical evaluation between the 10 N and 30 N torques, within each group and between groups.

Result of the general analysis of groups 1 and 2: The results, in mm (distance A–B), are listed below (Table 1) for the 10 Ncm and 30 Ncm values.

Groups 1 and 2 presented normal distribution of data after the Shapiro–Wilk test (*p* > 0.05), so the paired *t*-test was used.

### 3.1. Analog Results

**Figure 3:**
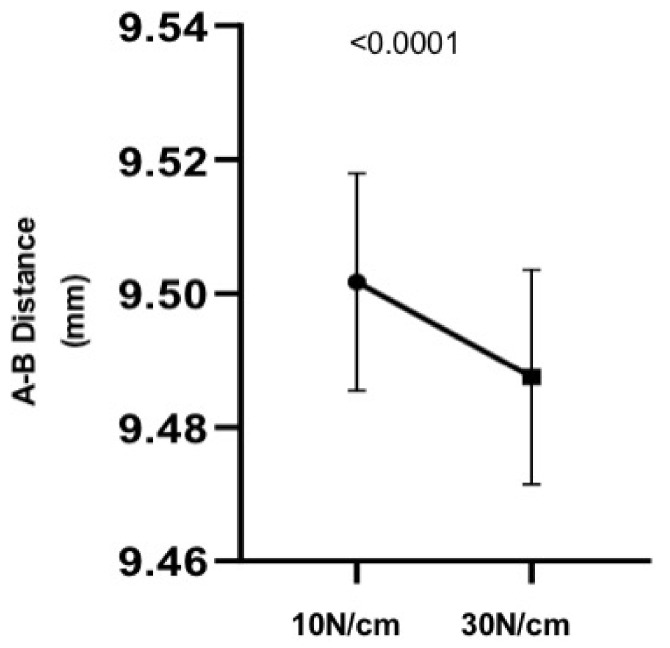
Chart showing a significant decrease in A–B distance between 10 N and 30 N in the pillars on the analogs (Group 1).

Considering the comparative analysis between the 10 Ncm and 30 Ncm torques in group 1 (analogs), it can be observed that there is a statistically significant difference between the distances A–B, with a greater distance in the 10 Ncm torque (Figure 3).

### 3.2. Implants Results

**Figure 4:**
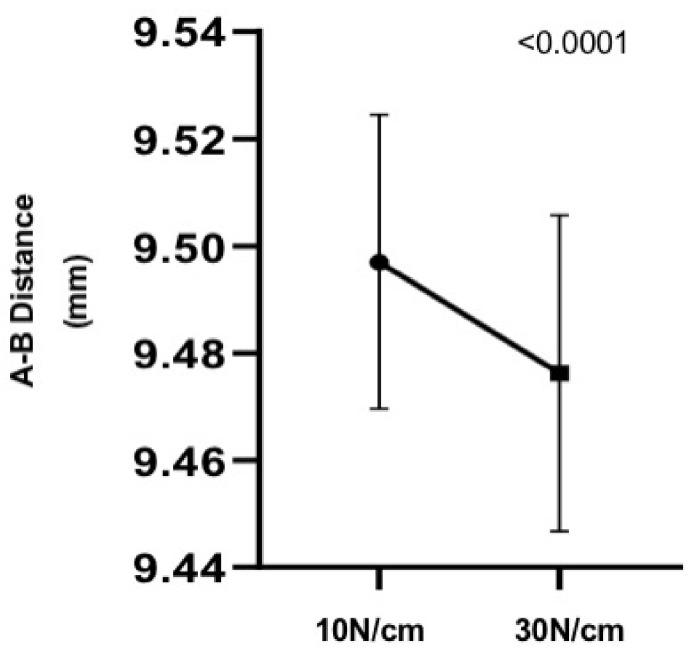
Chart showing a significant decrease in A–B distance between 10 N and 30 N in the pillars on the implants (Group 2).

Considering the comparative analysis between the 10 N and 30 N torques in group 2, it can be observed that there is a statistically significant difference between the distances A–B, with a greater distance in the 10 N torque (Figure 4).

### 3.3. 10 Ncm Analog and 30 Ncm Implant Comparison

**Figure 5:**
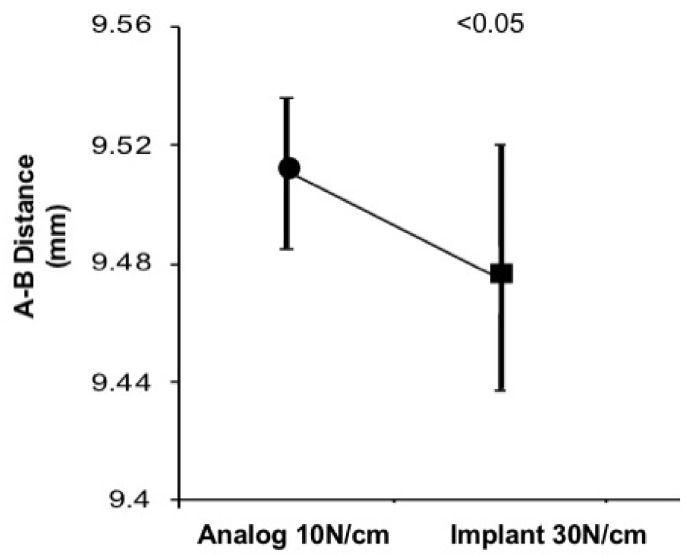
Chart showing significant decrease in A–B distance between 10 Ncm (analog) and 30 Ncm (implant).

In order to verify the clinical reproducibility of the experiment, comparisons between A–B measurements on the analog at 10 Ncm and implant at 30 Ncm were performed. The results showed a statistically significant difference, with greater A–B distance in the 10 Ncm torque in the analog (*p* < 0.05) (Figure 5).

### 3.4. 30 N Analog and 30 N Implant Comparison

**Figure 6:**
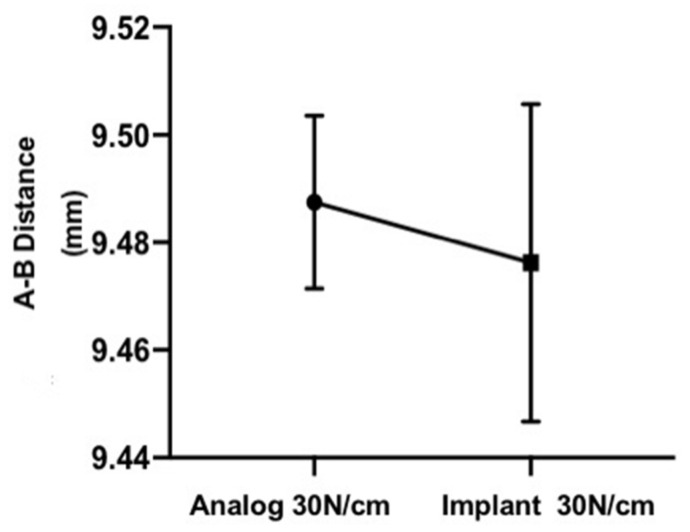
Chart showing the similarity between the distance A–B when the 30 N torque is applied to the analog and the implant.

In order to verify if the change in the laboratory protocol from 10 to 30 N could minimize the differences in the heights of the prosthetic abutments, the A–B distance between the analogs (30 N) and implants (30 N) was compared. There was no statistically significant difference between the groups for this torque (*p* > 0.05) (Figure 6). 

## 4. Discussion

Despite the long-term success associated with endosseous implant rehabilitation, complications related to mechanical and biological aspects still have high rates [9]. Mechanical failures represent about 60–80% of complications in implant dentistry [11], being one of the main problems related to fixed prosthesis failures. It is noteworthy that the discrepancy in the adaptation of prosthesis from the prosthetic laboratory to the adaptation in the patient’s oral cavity may be a decisive factor for the longevity of treatment. Therefore, this work aimed to evaluate the influence of manual torque force and the force recommended by the manufacturer on the total lengths of morse cone implant abutments. Our results showed that there is a difference in the heights of the prosthetic abutments when using the manual torque of 10 Ncm and the clinical torque of 30 N. The discrepant decrease in the distance between the implant platform to the top of the abutment was observed in both groups. However, when comparing prosthetic abutments supported on both analogs and implants with the same torque (30 Ncm), there was no difference in the height of the prosthetic abutment, which demonstrates that the difference in prosthesis height is related to the torque force and not to the torque component connected to the abutment.

The morse cone implants have a strong imbrication between the internal surface of the implant and the prosthetic component, leading to less movement between these structures and helping to prevent the passage of microorganisms from within the implant [12,13]. Therefore, in this research, the morse cone implant was selected to allow these results to be extrapolated to other types of connections that demonstrate greater prosthetic instability. However, our study showed that even in the presence of the characteristic clamping of this connection, when different torque forces are applied, a vertical displacement of the abutment can be promoted, and there can be possible space creation in the prosthesis components.

The presence of spaces between implant components is at great risk for the spread of a bacterial reservoir, accentuating possible peri-implant soft tissue inflammation and culminating in the development of mucositis [14,15], which is today characterized as a highly incident disease in the population rehabilitated with endosseous implants [16]. This disease is closely related to long-term success in implantology, as mucositis is the obvious precursor of peri-implantitis, which is a major cause of implant loss [17]. Thus, preventing the creation of prosthetic mismatches may be of great relevance to implant survival.

Another important aspect to consider is the torque applied to the prosthetic component of the implant. In theory, the torque should follow the manufacturer’s instructions. However, during fabrication of the prosthesis in the laboratory, the component is usually fitted over the analog, which simulates the implant by manual torque. Thus, two main problems can be generated: a lower torque during prosthesis making and prosthesis adaptation in an analog, not in an endosseous implant.

Al-Otaibi et al. [8] conducted a study comparing the effect of different applications of torque on implant-supported fixed unit prosthesis, showing that torsion may influence prosthesis adjustment and implant survival. Studies show that manual torque, often applied during prosthesis fabrication, reaches a maximum of 20 Ncm and has torque differences in about 48% of the prosthesis fabricated [18,19].

According to the study by Dellinges and Tebrock, in 1993 [2], screws that require more than 10 Ncm of torque cannot be tightened manually using commercially available hand wrenches. Therefore, mechanical or electrical torque control is required. In our study, all specimens were subjected to controlled torque forces in order to make the results replicable and standardized. We observed that the differences between the devices used in the laboratory during the making of the prosthesis, 10 Ncm, influence the difference in the height of the prosthetic abutment, which is the distance between the implant platform to the top of the abutment when it is subsequently submitted to the application of 30 Ncm torque.

On the other hand, the use of different torques on analogs and implants did not influence the height of the prosthetic abutment, showing that the use of the analog during the prosthesis fabrication is not directly related to future mismatches associated with the crown height but rather the applied torque. A study by Saber et al. [7] showed the discrepancy of the abutment height between the different types of implant platforms, showing greater discrepancy in the external hexagon system. However, in the present study, we did not consider the simulation of the laboratory phase of prosthesis confection, using analog, as done in our study.

The pattern of discrepancies observed in our study evidenced the decrease in the distance between the implant platform to the top of the abutment, similarly in all analog-analog, analog–implant, and implant–implant comparisons, when subjected to 10 Ncm and 30 Ncm torque. Comparing an “ex vivo” study to the patient’s mouth, the abutment in the mouth could generate significant changes in the height of the prosthesis, triggering infraocclusion and related pathologies. For this, future laboratory procedures may be developed, considering these discrepancies and their possible clinical consequences, aiming to minimize the damage to the peri-implant tissues. The presence of discrepancies in laboratory and clinical procedures shows that a correct torque is needed in the laboratorial procedures and then, another new screw is necessary to retain the crown in mouth in future protocols. In addition, future studies are needed, using different types of abutments and implant systems, in an attempt to ratify the applicability of the results found. In addition, the authors suggest exploring ways to minimize this discrepancy regarding its possible clinical issues.

## 5. Conclusions

This laboratory study concluded that there is a change in the distance between the implant platform to the top of the prosthetic abutment with different torques (*p* < 0.001), which is representative of the laboratory confection and clinical application of the unit prosthesis on morse cone implants.

## Figures and Tables

**Table 1:** Individual values of distances between points A and B (mm).

	10 Ncm	30 Ncm
Analogs (Group 1)	9.507	9.497
	9.497	9.479
	9.485	9.470
	9.515	9.498
	9.491	9.478
	9.511	9.494
	9.487	9.470
	9.503	9.505
	9.485	9.470
	9.536	9.514
Minimum value	9.485	9.470
Maximum value	9.536	9.514
Mean ± SD	9.5017 ± 0.016	9.487 ± 0.016
Implants (Group 2)	9.503	9.480
	9.487	9.468
	9.493	9.474
	9.463	9.437
	9.485	9.47
	9.466	9.441
	9.527	9.504
	9.488	9.462
	9.555	9.538
	9.503	9.488
Minimum value	9.463	9.437
Maximum value	9.555	9.538
Mean ± SD	9.497 ± 0.027	9.476 ± 0.029

## Data Availability

Data sharing is not applicable to this article.
